# Testing survey methodology to measure patients' experiences and views of the emergency and urgent care system: telephone versus postal survey

**DOI:** 10.1186/1471-2288-10-52

**Published:** 2010-06-09

**Authors:** Alicia O'Cathain, Emma Knowles, Jon Nicholl

**Affiliations:** 1 Medical Care Research Unit, School of Health and Related Research, University of Sheffield, Regent Street, Sheffield S1 4DA, UK

## Abstract

**Background:**

To address three methodological challenges when attempting to measure patients' experiences and views of a system of inter-related health services rather than a single service: the feasibility of a population survey for identifying system users, the optimal recall period for system use, and the mode of administration which is most feasible and representative in the context of routine measurement of system performance.

**Methods:**

Postal survey of a random sample of 900 members of the general population and market research telephone survey of quota sample of 1000 members of the general population.

**Results:**

Response rates to the postal and market research telephone population surveys were 51% (457 out of 893 receiving the questionnaire) and 9% (1014 out of 11924 contactable telephone numbers) respectively. Both surveys were able to identify users of the system in the previous three months: 22% (99/457) of postal and 15% (151/1000) of telephone survey respondents. For both surveys, recall of event occurrence reduced by a half after four weeks. The telephone survey more accurately estimated use of individual services within the system than the postal survey. Experiences and views of events remained reasonably stable over the three month recall time period for both modes of administration. Even though the response rate was lower, the telephone survey was more representative of the population, was faster and cheaper to undertake, and had fewer missing values.

**Conclusions:**

It is possible to identify users of a health care system using a population survey. A recall period of three months can be used to estimate experiences and views but one month is more accurate for estimating use of the system. A quota sample market research telephone survey gives a low response rate yet is more representative and accurate than a postal survey of a random sample of the population.

## Background

When patients in England need immediate advice or treatment for a health problem they can access a wide range of services. These services include 999 ambulance, emergency departments, general practice (also known as family practice internationally), pharmacy, NHS Direct the 24 hour nurse-led telephone helpline, walk-in centres, and minor injury units. Policy makers see these services as an emergency and urgent care system,[1] and promote integrated care across all services[2]. Patients want these services to work as a system,[3] and need coordinated services because they often use two or more services in the process of obtaining definitive care for an urgent problem[4].

Commissioners and providers of care are interested in the quality and outcomes of care experienced by patients[5]. Standard questionnaires are available to measure patients' experiences and views of individual services, for example in-hours general practice,[6] out of hours primary care,[7] 999 ambulance emergency services,[8] and emergency departments[9]. Although a questionnaire has been developed which addresses the interface between secondary and primary care [10] rather than a single service, there are no standard questionnaires which measure patients' experiences and views of a system. We developed and tested a questionnaire to measure patients' experiences and views of the emergency and urgent care system, one of a number of 'systems' that exist within the National Health Service (NHS)[11]. Below we explore three methodological challenges we faced when considering how best to undertake a survey of a system rather than a single service.

### 1. Identifying users of the emergency and urgent care system

Monitoring the performance of services from the patient perspective usually involves a survey of recent users, for example callers to NHS Direct, or attendees at a walk-in centre. The administrative records of services can be used to identify recent users. However, the system is a virtual entity and does not keep records of its own. There are two possible approaches for identifying users of a system rather than an individual service. First, it would be possible to access the records of all component services within a system. This would raise intractable problems due to the large number of services which make up the emergency and urgent care system,[4] and the problems of record linkage, sampling and double counting. Furthermore, it excludes anyone who attempted, but failed, to use the system. Second, it would be possible to screen the general population for recent users and follow this with a survey of those recent users. The use of screening questionnaires in a two-stage survey approach has been used in other substantive areas of research [12-15]. A population screening questionnaire has been administered either by post or telephone and then relevant respondents contacted again by telephone, post or interview to complete a lengthier, more detailed questionnaire. Screening postal questionnaires have obtained response rates of 49%[13] and 67%[12] for example, and a telephone screening questionnaire obtained 52%[15]. Response rates to the second stage questionnaires in these studies varied between 58% and 87%. In the context of a survey of the emergency and urgent care system, the two stage approach could introduce an unacceptable delay between first contact with a respondent and obtaining details about their recent use of the system. Delays may increase recall bias and possibly cause confusion if a further episode of system use takes place between the screening questionnaire and the follow-up. Therefore a combined questionnaire, covering screening for an event, and details of any event, might be more appropriate. Indeed we used this approach successfully in a previous population survey of the use of unscheduled care [4].

The screening question is a key aspect of a population survey approach. The general population must understand what is meant by the term 'emergency and urgent care'. Although people have a clear and consistent understanding of the term 'emergency', the meaning of 'urgent' is problematic [3]. Focus groups of people who had recently used services within the system identified the importance of offering examples of the range of services which might be accessed for urgent care, as well as offering a definition of the term.

### 2. Selecting an appropriate period of recall of events

A survey of recent users of a service requires respondents to remember the detail of their last event. However, a population survey which screens for recent system users requires first that the respondent recalls whether a health event occurred in a particular time period, and then recalls details of the last event. Psychologists have studied autobiographical memory[16]. For non-threatening issues, memory errors represent the greatest problem[17] either through 'episode omission' whereby the respondent does not recall the event which occurred in the specified time period, or by 'episode telescoping' and 'episode expansion' whereby the respondent puts the event more recently in time or more distant in time than it really was. Different recall periods for health events have been used, in particular four weeks,[18] eight weeks,[18] three months,[19,20] six months,[21,22] and one year[18,19,23]. No consistent results have emerged from previous research about the optimum recall period for different health events. However, it is clear that recall is never perfect and that it can depend on the severity or significance of the event,[24] the clarity of definition of the event,[19] the types of details asked for, and the characteristics of the population under study[16].

Events within the system under study here can vary widely in their severity, from minor actions such as going to a pharmacist, to major actions such as calling a 999 emergency ambulance. A short recall period will facilitate memory but limit the numbers of respondents who had an event and the number of rare events captured. The optimum recall period in a system survey needs to be long enough to include a large number of events without unduly affecting recall of event occurrence or details of any events.

### 3. Measuring the patient experience in a feasible and representative way

Measurement of the patient perspective of the emergency and urgent care system can be undertaken as part of a research study. However, it is also important that those responsible for managing systems are able to routinely monitor the quality of their system and assess the effect of changes they make to their local systems. It must be feasible for health care commissioners to undertake a survey of a system quickly and easily, and in a way which is representative of their local population. Health care commissioners may not have in-house facilities to undertake large population surveys and therefore the use of market research companies may be a more feasible option. Market research companies can use postal or telephone surveys for population surveys. When using the latter they tend to use random digit dialling with quota sampling[25-27]. The low response rates associated with this approach can lead to a lack of credibility. Concerns include that a survey is not representative of the population characteristics which have not been included in the quota sampling, and that there may be bias in estimates of variables of interest.

Our aim was to address these methodological challenges, testing the feasibility of a population survey to identify system users, the optimum recall period to use for maximising the numbers of system users identified while minimising recall bias, and the most feasible and representative administration of a survey undertaken in the context of routine performance management within the NHS.

## Methods

We undertook two types of survey: a random sample postal survey of the general population and a quota sample telephone survey of the general population. We undertook both surveys within an emergency and urgent care system managed by one group of commissioners and service providers. The system covered services commissioned by two primary care trusts for a population of one million people. We obtained ethical approval from a local NHS Ethics Committee, and research governance from the two primary care trusts.

### Postal population survey

Our intention was to undertake a 'gold standard' postal survey of 1000 members of the general population to identify recent users of the emergency and urgent care system. We wanted to include all ages within the population because children are frequent users of services in this system[4]. We considered identifying the general population sample using the electoral register. However, this includes adults only, and people have the option of removing their names and addresses from the publicly accessible register. We therefore used general practice lists to identify members of the general population. We planned to select a stratified random sample of 20 practices and a random sample of 50 patients from each practice to obtain a total of 1000. Stratification was by area to ensure geographical representation across the whole population. In 2007 we selected 20 practices and contacted the named practice manager in each, requesting participation in the research. Practices which did not want to participate were replaced by other practices from the same geographical sampling stratum. In total we contacted 65 practices - approximately half of all the practices in the network - and 13 agreed to participate. When we realised how difficult it was to recruit practices we increased our request from a sample of 50 patients to a sample of 100 patients from practices recruited later in the process. The 13 practices selected a random sample of patients from their lists, removing people from the sample to whom they felt it would be inappropriate to send a postal questionnaire. Questionnaires were sent to adults aged 16 and over, and the parent/guardian of children aged up to 16 years of age. The practices posted questionnaires on behalf of our research team and respondents returned completed questionnaires directly to our university via reply paid envelopes. Two reminders were sent to non-respondents by the general practices. Practices were paid for administrative time and postal costs were provided.

### Telephone population survey

We engaged a market research company to undertake a telephone survey of a random sample of the general population. Our plan was that they would identify 1000 telephone numbers at random and undertake up to four attempts to contact the owner of the telephone number. However the market research company did not normally undertake surveys in this way. Their standard approach was to call telephone numbers once only until they obtained 1000 respondents who fitted the age sex profile of the population, that is, quota sampling. We decided to adopt this market research company approach in the interest of the feasibility of health care commissioners undertaking such a survey routinely. We identified the postcodes covering the system population and supplied them to the company. They undertook random digit dialling of landline numbers within these postcode areas with one attempt to contact a number. Their aim was to identify 1000 respondents fitting the population profile in terms of age and sex. The person answering the telephone was asked if they were over 16 years old. If they were, they were asked to complete the questionnaire, and if they were not, then they were asked if the interviewer could speak to someone in the household over 16. Once an adult was identified, the ages of children in the household were identified. The adult or a child was selected as the focus of the interview in line with meeting the quota sample. The assumption was made that any adult answering on behalf of a child knew about that child's use of health care.

### Questionnaire

The same questionnaire was used in both surveys. It was developed based on qualitative research with recent users of the system[3]. All participants were asked a screening question about use of urgent care and some socio-demographic questions. If they had attempted to contact emergency or urgent care services in the previous three months they were asked to complete the remaining parts of the questionnaire about the most recent event. They described the first three services used in their most recent event and answered 22 satisfaction items about the system. The telephone version was adapted to ensure it worked in the context of a telephone interview: the list of services used in the most recent contact with the system was shortened from 20 options to the 10 most commonly used services so that the interviewee would not have to listen to a long list. An introductory script was written to replace a covering letter. A section was added to identify children within households. Both surveys were administered in English only. A copy of the postal questionnaire is available in Additional File 1.

### Routine data to test representativeness of the population surveys

We obtained 2001 Census data about the population covered by the system in order to consider the representativeness of the population surveys. The health care commissioners provided us with routine data on numbers of their residents using specific services in their system during 2006/7. We compared routine data on utilisation of services with data from the population surveys to consider the accuracy of estimates of system utilisation from the population surveys.

### Analysis

Recall was studied by plotting the proportion of respondents reporting that they attempted to use the system by the week in which the most recent contact was made. Accuracy of rates of service use was tested by comparing reported rates in the surveys with the gold standard rates based on routine data. Consistency of reports of events with different lengths of recall was tested by comparing experiences and views of events occurring within four weeks of recall with those occurring beyond four weeks. Response rates and representativeness were compared for the two modes of administration of the population survey. Chi-squared tests and t-tests were used to compare proportions and continuous variables respectively. The postal population survey was analysed at the individual level, without taking the clustered nature of the data into consideration. Data were analysed using SPSS version 12.1.

## Results

### Response rates

For the postal population survey, 13 practices of the 65 approached agreed to participate. The first eight practices randomly sampled 50 patients on their lists, and the next five sampled 100 people, giving a total sample of 900 people. The survey was administered between September and December 2007. Each practice took six weeks to gather data because two reminders were sent at two-weekly intervals. Practices sent the survey at different times in this period due to difficulty coordinating all practices to send the questionnaire at the same time. When 'return-to-senders' were removed from the denominator, the response rate was 51% (457/893). The telephone survey was undertaken in one week in July 2007. Out of 18091 calls made, 4871 numbers were unobtainable, and there was no response to a further 8689 because the telephone was unanswered (3806), engaged (320), on answer-phone (3074) or the person was not available (1489). Some people refused to respond to the questionnaire (2221), belonged to age-sex groups where the quota was already filled (1272), or were duplicates (24). The response rate was 8.5% (1014/11924) from people who were contactable and eligible for inclusion. The 1000 respondents fitting the telephone survey quota sampling were selected for analysis.

### Identification of system users

Both surveys were able to identify system users, although the proportion of users identified differed by mode of administration: 25% (113/457) and 15% (151/1000) of respondents reported seeking urgent health care in the previous three months in the population postal and telephone surveys respectively. In the population postal survey some respondents ticked 'yes' to the urgent care question but reported their most recent event at over 13 weeks. When they were excluded, the postal survey event occurrence reduced to 22% (99/457). That is, the postal population survey identified a higher proportion of people reporting recent use of the system than the telephone survey.

### Recall of event occurrence

**Figure 1:**
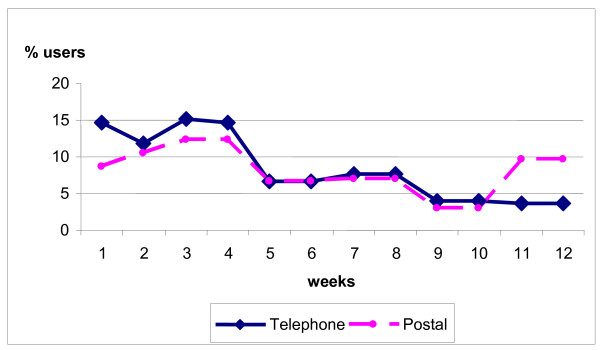
Percentage of all users reporting use by week of recall period*. *use for weeks 5 and 6 is half of total use reported for weeks 5 and 6 combined, and similarly for weeks 7-8, 9-10 and 11-12.

When reporting the timing of their most recent event, respondents expressed digit preference after week 4, for example, reporting one 8 and 12 weeks ago rather than 7 and 11 weeks respectively. Therefore we smoothed the distribution of respondents' most recent events over time by averaging total reported use for weeks 5-6, 7-8, 9-10, 11-13. We plotted the smoothed distribution of the most recent events for each week over the three month period (Figure 1). The lines on Figure 1 represent the distribution of all reported events by the week in which they occurred. A uniform distribution would indicate similar recall of event occurrence over the time period. There was evidence, from both the telephone and postal surveys, of a reduction in recall of event occurrence after 4 weeks, with further problems emerging after 8 weeks. There was also evidence of episode telescoping in the postal survey, with respondents placing more distant events into the three month time frame to make the questionnaire more relevant to them. The sample size was too small to plot recall for individual services within the system. The estimated use of the system in a four week recall period was 10% (46/457) for the postal survey and 8.5% (85/1000) for the telephone survey.

### Accuracy of recall of event occurrence

Given concerns about the reduction in recall of event occurrence after four weeks, we used the survey data for events occurring within four weeks to estimate the population use of individual services. We multiplied the number of most recent events by the mean number of events which respondents reported in that four week period: a mean of 1.5 in the postal survey and 1.8 in the telephone survey. We calculated rates of use per 1000 population in a four week period for each service in the system. The telephone population survey appeared to be more accurate than the postal survey in determining use of specific services, with the exception of walk-in centres (Table 1).

**Table 1:** Rate per 1000 population per month using specific services in the system

Service	Postal survey	Telephone survey	Routine data
GP out of hours	26	13	13
A&E	23	11	12
999	3	7	6
Urgent care centres (MIUs)	6	4	5
WIC	3	9	0.5

### Recall of experiences and views

We tested whether respondents' experiences and views were dependent on the length of recall of their most recent event. We compared experiences and views for events occurring within four weeks of completing the questionnaire, and events occurring between four weeks and three months. We did this separately for the postal and telephone surveys in case reporting differed by mode of administration. Statistical power was low due to small numbers. There were some statistically significant differences by time period for key variables (Table 2), although there was no consistent pattern concerning superiority of either administration mode. Of the 22 satisfaction items, only one was statistically significantly different for events recalled within 4 weeks compared with events beyond 4 weeks.

**Table 2:** Comparison of experiences and views of most recent event by recall period of event

	Postal <= 4 weeks N = 46	Postal 5-13 weeks N = 52				Telephone <= 4 weeks N = 85	Telephone 5-13 weeks N = 66
Mean number of services involved in most recent event	2.3	2.6				1.9	2.2
		P = 0.294					P = 0.071

When help was sought from first service+							
In hours	67%	77%				82%	69%
Out of hours	33%	23%				18%	31%
		P = 0.286					P = 0.075

Case managed with sufficient urgency+							
Definitely not/No, I don't think so	22%	15%				11%	12%
Yes, I think so	20%	46%				22%	23%
Yes, definitely	59%	39%				67%	65%
		P = 0.021					P = 0.951

Overall rating of care received+							
Excellent	30%	31%				52%	33%
Very good	30%	35%				26%	44%
Good-very poor	39%	35%				22%	23%
		P = 0.875					P = 0.039

+response categories collapsed to ensure validity of chi-squared test

### Representativeness

The response rates to the two population surveys were very different. We compared the socio-demographic profile of the two population survey respondents with the census population from which they were sampled (Table 3). People below the age of 44 years old were underrepresented and people aged over 45 were overrepresented in the postal survey; males were also underrepresented. In contrast the telephone survey was designed to be, and therefore was, representative of the age and sex structure of the population through quota sampling. It was also representative of ethnic minority groups whereas the postal survey was not. The postal survey was superior only in terms of representing home ownership; the telephone survey overrepresented people who owned their homes.

**Table 3:** Socio-demographic profile of survey respondents compared with population

	Postal sample N = 457 %	Telephone sample N = 1000 %	2001 census population %
Age			
< 5	2	5	5
5-9	5	6	6
10-15	5	6	6
16-24	7	11	12
25-34	6	11	12
35-44	13	16	16
45-54	19	14	13
55-64	20	13	13
65+	24	17	17

Sex			
Male	42	50	49
Female	57	50	50

Ethnic group			
White	98	95.8	96.0
Asian	0.5	2.7	2.3
Other	1.6	1.5	1.7

Accommodation type*			
Owner	76	84	73
Rented/other	24	16	27

*Home ownership does not compare like with like: it was measured for individuals in our surveys and for households in the census

### Feasibility of population surveys

The telephone survey was undertaken by a market research company, analysed and reported, all within one week. The postal survey took months in terms of recruiting general practices and sending up to two reminders to respondents. The cost of the telephone survey was approximately £10,000 (2007 prices) to identify 150 system users in the previous three months. The cost of the postal population survey was higher when costs of printing, postage, researcher and administrative time were summed. A conservative estimate was £12,000 to identify 99 users of the system. The amount of missing data within the postal questionnaire varied by item but was always higher than rates of missing data for the telephone survey[11].

## Discussion

### Summary of findings

It is feasible to identify system users using a population survey. Although the telephone survey used market research quota sampling and obtained a low response rate of 8.5%, it appeared to perform better than the postal survey in terms of representativeness by age, gender and minority ethnic communities, and estimating use of different services in the system. It also cost less and suffered less from missing values.

A recent randomised controlled trial of a postal versus telephone survey did not identify one mode of administration as superior[28]. This supported an earlier review of surveys in health care which identified four randomised controlled trials of postal and telephone surveys, revealing little consensus about the benefits of one over the other [29]. However a higher rate of missing values was found in the postal survey for both the recent trial [28] and the one trial in the earlier review which measured this[29]. This supported our findings. There is some evidence that telephone surveys can elicit more extreme responses and more positive responses than postal surveys,[28] although this is by no means a consistent finding [29]. This is an important issue to bear in mind if telephone surveys are to be recommended for obtaining the patient perspective of the emergency and urgent care system. However, it is less important when using telephone surveys to monitor patient views over time within systems because the focus would be changes in values rather than absolute values.

Use of the system was estimated as 10% or 8.5% in a four week period for the postal and telephone surveys respectively. Other researchers have estimated use of urgent care in a month to be 23% of adults seeking care for themselves or someone else, with the figure rising to 56% when the time period was the previous year[30]. Use of unscheduled care in the previous four weeks, which is similar to urgent care, has been estimated at 16%[4]. Our estimates are smaller than those found elsewhere but we did validate reported use of some services in the system and our estimates appeared to be accurate. Four weeks appears to be an accurate recall period for estimating event occurrence but limits the number of events identified. Three months underestimates utilisation of the system but offers more events for users to describe their experience and views. We have shown that there is little difference in the experiences and views of events described in the early and late recall periods and therefore recommend that a three month period of recall is used to assess experiences and views of the system.

The telephone survey cost £10,000 to identify 150 users of the system, that is, £67 per user. Although this is a large cost per user, the telephone survey methodology described here offers an unbiased approach to identifying a comparable group of people over time, thus allowing health care commissioners to monitor changes in their system over time.

### Biases in postal and telephone surveys

Bias arises when non-response in a survey is related to the outcome being measured. Here, the most important outcome is satisfaction with the system. The potential for non-response bias differed for the postal and telephone population surveys. For the postal survey the potential for bias was introduced by the exclusion of general practices unwilling to participate, the exclusion of those not registered with a general practice or registered but recently moved, the screening of individuals' names by health professionals for those who might be distressed by the contact (this might include frequent users of the urgent care system such as people with mental health problems), the likely exclusion of people with literacy problems, and low saliency of the questionnaire for the majority of people (non-users of the system). For the telephone survey the potential for bias was introduced by the exclusion of those without telephones or landlines, those who screened their telephone calls, and people who do not spend much time at home to receive telephone calls.

A comparison of a postal survey with a probabilistic random digit dialling telephone survey in the United States identified some advantages of the telephone approach in health research[31]. The telephone survey had higher proportions of respondents from ethnic minority communities and less educated groups, and a lower proportion of missing values. This supports our findings about the representativeness of the telephone survey. The United States study also found problems with the telephone survey which have been identified previously in some literature, namely lower response to sensitive questions and use of extremes of response sets to offer socially desirable answers. Our questionnaire did not contain any obviously sensitive questions, for example we did not ask for details of the health problem for which help was sought apart from whether it was an illness, injury or other type of problem. However, questions about health and health care may be perceived as sensitive by respondents and thus the telephone survey may have been affected by this. It is possible that there was social desirability bias due to respondents worrying whether their use of health services appeared to be appropriate, and concerns about not being seen to complain about health services. These problems are less problematic if telephone surveys are used to measure change over time rather than prevalence of behaviour or attitudes. However, these problems must be borne in mind when interpreting survey findings.

Interestingly, the United States study described above identified that the 6% of postal survey responders who were living in households without a landline, that is, used mobile phones only, had health behaviours different from those with landlines. They were more likely to participate in risk taking behaviour for HIV. This bias may be context specific to a survey undertaken in 2005 in the United States but raises the important concern that the prevalence of mobile phone only households may increase over time in the United Kingdom and thus exclude people from our telephone survey. In the United Kingdom, ownership of landlines fell from 94% to 89% of households between 1997 and 2007, while 78% of households had mobile phones[32]. We could not find any UK data on mobile only households, especially the age and socio-economic groups most likely to be without landlines. However, young people, and students in particular, might be more likely to live in households without landlines than other subgroups of the population. Telephone survey methodology will need to address this potential exclusion in the future.

### Strengths and limitations

The strength of this study is that different approaches to methodology have been tested empirically. Limitations include the low power for some of the statistical comparisons made, the low response rate of the telephone survey although this is typical of these types of surveys,[25-27] the use of English only versions of both surveys so that those who could speak English well enough for the telephone survey and read and write it well enough for the postal survey were included, and the potential for 'primacy' effects of selection of the response options nearer the beginning of any list to operate in the postal questionnaire while 'recency' effects of selection of the response options closer to the end of the list operated in the telephone survey[33]. Finally, population estimates were based on the 2001 Census and the demographics of the area could have changed by 2007.

The postal and telephone surveys were undertaken at different times - the telephone survey was undertaken in July, covering system use in a three month period between April and June, and the postal survey between September and December with the three month period lying between June and November. There is seasonal variation in the use of some services in the system. In particular, general practice consultations for respiratory and influenza-like symptoms, and pressures on emergency hospital beds, increase enormously in the winter months of December to February while remaining steady in other months. This peak use of services was not included in either the telephone or postal survey recall periods but part of it was likely to have affected the postal survey and this could account for some of the higher reporting of system use in this survey. It is also the case that the routine data which the survey were compared with covered the whole period of 2006/7 which would include the winter peak. Because of this we would expect both the telephone and postal survey to underestimate average system use because they did not include the winter peak.

### Implications of using the telephone survey

The telephone survey does not make use of NHS sampling frames and therefore does not need approval from an NHS Ethics Committee in the UK. However if it is used for research purposes then ethics approval must be sought. We have used it since this study and sought ethics approval from our university. If the survey is used by those managing an emergency and urgent care system then this would be classified as service evaluation and would not need NHS ethics committee approval in the UK.

It is important to bear in mind the limitations of the telephone survey in terms of excluding mobile phone only households and homeless people. It also assumes that adults completing the survey on behalf of a child know about that child's use of health care. We tested an English only version of the survey but some market research companies offer a translation service for telephone administered surveys. This is a useful approach so that people who do not speak English - and who may have difficulties using the emergency and urgent care system because of this - can be included in the survey.

## Conclusions

It is possible to identify users of the emergency and urgent care system through a population survey. A recall period of three months can be used to estimate experiences and views, but a recall period of four weeks is needed to estimate use of the system. A standard market research telephone survey using quota sampling gives a low response rate yet is superior to a postal survey of a random sample of the population because it is more representative and is feasible for commissioners of health care systems.

## Competing interests

The authors declare that they have no competing interests.

## Authors' contributions

AOC contributed to the design of the study, undertook the analysis, wrote the first draft of the manuscript and approved the final version. EK contributed to the analysis and interpretation of the data, edited drafts of the manuscript and approved the final version. JN conceived the idea, designed the study, contributed to analysis and interpretation of the data, edited drafts of the manuscript, and approved the final version.

## Pre-publication history

The pre-publication history for this paper can be accessed here:

http://www.biomedcentral.com/1471-2288/10/52/prepub

## Supplementary Material

Additional file 1 **Questionnaire**. Copy of postal questionnaire used in the study. Click here for file
