# Supplementary material for: Testing survey methodology to measure patients' experiences and views of the emergency and urgent care system: telephone versus postal survey
Source: BMC Med Res Methodol. 2010 Jun 9;10:52. doi: 10.1186/1471-2288-10-52 (PMC2905427; doi:10.1186/1471-2288-10-52)
Supplement: Additional file 1 — Questionnaire. Copy of postal questionnaire used in the study. [file 1471-2288-10-52-S1.DOC]

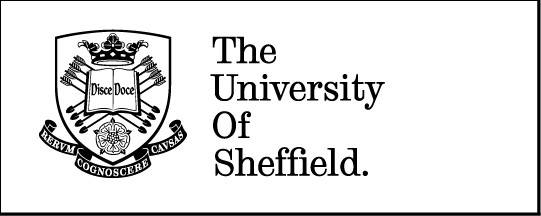


|  |  |  |  |  |
| --- | --- | --- | --- | --- |

**CONFIDENTIAL**

**University of Sheffield**

**Medical Care Research Unit**

**Seeking help for urgent health problems**

| We want to know the views of people who have tried to get help from services such as a GP, nurse, pharmacist, accident and emergency department, 999 ambulance, or a telephone advice line, for a health problem where you felt help or advice was needed on the same day. We hope you will help. Completing this questionnaire will probably take 15 minutes.  If you have not needed help urgently we would still like you to answer the questions on the first page. This will only take a minute.  The information you give will help to plan future health services. Please tick the appropriate boxes. | | | | | | | | | | | | |
| --- | --- | --- | --- | --- | --- | --- | --- | --- | --- | --- | --- | --- |
| **1.** Please enter today’s date | / / *(day/month/year eg 7/3/07)* | | | | | | | | | | | |
|  |  | | | | | | | | | | | |
| **2.** Is it your name on the envelope? | | | | | **YES** | | | **NO** | | | | |
| If **‘NO’**, are you the | | | | |  | | | | | | | |
| Parent or guardian of the person | | | | | |  | | |  |  |  |  |
| Other relationship | | | | | |  | | |  |  |  |  |
|  | | | |  | | | | | | | | |
| **Please fill out questions** **3-8 below**. Remember to answer only for **the person named on the envelope. E.g.** if completing Question 4 for a child, the answer to ‘Age’ is the child’s age. | | | | | | | | | | | | |
| **3. In the last 3 months** has help been sought for **an urgent health problem**? *This includes trying to contact a service such a GP, accident and emergency, chemist, 999 ambulance, dentist etc where you felt that help or advice was needed on the same day.*    **YES** **NO** | | | | | | | | | | | | |
| If **‘YES’**, please fill out the rest of this questionnaire | | | | | | | | | | | | |
| If **‘NO’**, could you please fill out questions 4-8 only on this page? This information helps us to understand the different kinds of people who respond. THANK YOU | | | | | | | | | | | | |
|  | | |  | | | | | | | | | |
| **4. Age** | | **5. Sex** Male Female | | | | | | | | | | |
|  | |  | | | | | | | | | | |
| **6. Ethnic group** | |  | | | | | | | | | | |
| White  Black or Black British  Asian or Asian British | | Mixed  Chinese  Other ethnic group please say_______________ | | | | | | | | | | |
|  | | |  | | | | | | | | | |
| **7. Accommodation** | | |  | | | | | | | | | |
| Owner occupied/mortgaged | | | Rented or other arrangements | | | | | | | | | |
|  | | |  | | | | | | | | | |
| **8**. **First part of post code** e.g. DE5 _____________ | | | | | | |  | | | | | |
|  | | | | | | | | | | | | |

|  |  |  |  |
| --- | --- | --- | --- |
| **SECTION A. Seeking help for an urgent health problem in the last 3 months. Please think about the most recent time that help was needed** | | | |
| **A.1 Now, thinking about the most recent time help was needed urgently, how many weeks ago was that?**  weeks | | | |

|  | | | |
| --- | --- | --- | --- |
| **A.2 Again, thinking about the most recent time, how long after thinking this health problem was urgent was help sought? *Please tick one*** | | | |
|  | |  | |
| Immediately |  | Between 12 and 24 hours |  |
| Less than 2 hours |  | More than 24 hours |  |
| Between 2 and 12 hours |  |  | |
|  |  |  | |
|  | | | |
| If ‘More than 24 hours’, how long? hours | | | |
|  | | | |

| **A.3 What type of health problem was it?**  ***Please tick one*** | |
| --- | --- |
| Illness | please say what ___________________________ |
| Injury | please say what ___________________________ |
| Other | please say what ___________________________  *(If you feel that you do not want to give details then please leave this blank)* |

|  | | | |
| --- | --- | --- | --- |
| **A.4** Still thinking about the most recent health problem, please tick the services that were involved in giving help or advice. Include all those you tried to contact, **even if this was not successful.** | | | |
|  | | | |
| ***Please tick all those involved*** | | | |
|  | |  | |
| Dentist |  | Minor Injuries Unit |  |
| Chemist (for a prescription) |  | Walk-in Centre |  |
| Chemist (for treatment or advice) |  | Hospital A&E department |  |
| Family doctor (GP) from my usual practice |  | A hospital clinic or day ward |  |
| Someone at my usual practice but  not a doctor |  | Admission to hospital over night or longer |  |
| GP out of hours/GP emergency service |  | Physiotherapist |  |
| NHS Direct |  | Social services |  |
| Other telephone helpline |  | Mental health crisis team |  |
| Voluntary service/ charity |  | Occupational Health |  |
| Complementary or alternative practitioner |  | 999 emergency ambulance |  |
| If another service was involved that is not listed above, please write them below: | | | |
| _________________________________________________ | | | |

| **A.5** How many **services** were involved altogether?  For example, if you saw the GP, a chemist for a prescription, went back to see the GP again, and then went to a hospital clinic for the same health problem this would be **4** services. | | |  |
| --- | --- | --- | --- |
|  |  |  | |

| You may have tried to contact one or more services. We would like to know about each service, starting with the first one you tried to contact. |
| --- |

| **SECTION B. The first service** | | | |
| --- | --- | --- | --- |
|  | | | |
| **B.1** What was the **first** service? | | | |
| ***Please tick one*** | | | |
| GP in hours |  | Walk-in Centre |  |
| GP out of hours |  | Minor Injuries Unit |  |
| Accident and Emergency |  | A pharmacist or chemist |  |
| 999 Emergency Ambulance |  | NHS Direct |  |
| Mental health crisis team |  |  |  |
| If the **first** service involved is not listed, please write it here | | | |
| ___________________________ | | | |
|  | | | |

|  |  | | | |
| --- | --- | --- | --- | --- |
| **B.2 When was help sought?** | | | | |
| ***Please tick one*** | | | | |
| Monday to Friday between about 8.30 am and 6.00 pm | |  |  |  |
| Monday to Friday outside these hours | |  |  | |
| Saturday or Sunday, any time | |  |  | |
|  | | |  | |

|  | | |  | |
| --- | --- | --- | --- | --- |
| **B.3 How was help sought?** | | |  | |
| ***Please tick one*** | | | | |
| By telephone |  |  | |  |
| By the Internet |  | |  | |
| In person |  | |  | |
| If how help was sought is not listed, please write it here ____________________________ | | | | |
|  | | | | |

| **B.4** Did contact with this **first** service cost money, for example, on phone calls, petrol, bus fares, parking,  taxi fares etc? | **YES** | **NO** |
| --- | --- | --- |
|  |  | |
| If **‘YES’**, approximately how much did it cost? | £ : p | |
|  |  | |
|  |  | |

| **B.5** What help did this **first** service give? | | | | | | | | |
| --- | --- | --- | --- | --- | --- | --- | --- | --- |
| *Please tick* ***all*** *that apply* | | | | | | | | |
|  |  | |  | | | |  | |
| Treatment |  | | Physical examination | | |  | | |
| Tests |  | | Advice to go to another service | | |  | | |
| Prescription |  | | Contacted another service for you | | |  | | |
| Information |  | | Asked you to come back again | | |  | | |
| Reassurance |  | | Nothing | | |  | | |
|  | | | | | | | | |
| If what was given is not listed, please write it here ________________________________ | | | | | | | | |
|  | | | | | | | | |
|  | | | | | | | | |
|  | | | | | | | | |
| **B.6** After this first service did you try to contact another service to seek help or advice about the same problem? | | | | | **YES**  **NO** | | |  |
|  | | | |  | | | | |
| If ‘**YES**’, why did you take this action? | | | | | | | | |
| *Please tick* ***all*** *that apply* | | | | | | | | |
| I was told to do so by the first service | |  | | The health problem got worse | | | |  |
| I wanted another opinion | |  | | The health problem changed | | | |  |
| I could not get access to the first service | |  | | I was not satisfied with the response from the first service | | | |  |
| If your reason(s) are not listed, please write them here | | | | ______________________________________ | | | | |
|  | | | |  | | | | |

|  |
| --- |
| If another service was involved **or** you went back to the first service again, please answer the questions in **Section C.** |
|  |
| If no-one else was involved, please go to **Section E** on **page 9.** |
|  |

| **SECTION C. The second service** | |  | |
| --- | --- | --- | --- |
|  | |  | |
| **C.1** What was the **second** service? | | | |
| ***Please tick one*** | | | |
| GP in hours |  | Walk-in Centre |  |
| GP out of hours |  | Minor Injuries Unit |  |
| Accident and Emergency |  | A pharmacist or chemist |  |
| 999 emergency ambulance |  | NHS Direct |  |
| Mental health crisis team |  |  |  |
| If the **second** service is not listed, please write it here __________________________ | | | |

|  |  | | | |
| --- | --- | --- | --- | --- |
| **C.2 When was help sought?** | | | | |
| ***Please tick one*** | | | | |
| Monday to Friday between about 8.30 am and 6.00 pm | |  |  |  |
| Monday to Friday outside these hours | |  |  | |
| Saturday or Sunday, any time | |  |  | |
|  | | |  | |

|  | | |  | |
| --- | --- | --- | --- | --- |
| **C.3** How was help sought? | | |  | |
| ***Please tick one*** | | | | |
| By telephone |  |  | |  |
| By the Internet |  |  | | |
| In person |  |  | | |
| If how help was sought is not listed, please write it here _______________________ | | | | |
|  | | |  | |

|  | | | |
| --- | --- | --- | --- |
| **C.4** Did contact with this **second** service cost money,  for example on phone calls, petrol, bus fares, parking, taxi fares, etc. | | **YES** | **NO** |
|  | | | |
| If ‘**YES**’, approximately how much? | £ : p | | |
|  |  | | |

| **C.5** What help did this **second** service give? | | | | |
| --- | --- | --- | --- | --- |
|  | | | | |
| *Please tick* ***all*** *that apply* | | | | |
|  | | | | |
| Treatment |  | Physical examination | |  |
| Tests |  | Advice to go to another service | |  |
| Prescription |  | Contacted another service for you | |  |
| Information |  | Asked you to come back again | |  |
| Reassurance |  | Nothing | |  |
|  | | | | |
| If what was given is not listed, please write it here | | | | |
|  | | | __________________________________ | |
|  | | |  | |
|  | | |  | |

|  | | | | |
| --- | --- | --- | --- | --- |
| **C.6** After this second service did you try to contact another service to seek help or advice about the same problem? | | | **YES**  **NO** |  |
|  | |  | | |
| If ‘**YES**’, why did you take this action? | | | | |
| *Please tick* ***all*** *that apply* | | | | |
| I was told to do so by the second service |  | The health problem got worse | |  |
| I wanted another opinion |  | The health problem changed | |  |
| I could not get access to the second service |  | I was not satisfied with the response from the second service | |  |
| If your reason(s) are not listed, please write them here | | ______________________________________ | | |
|  | |  | | |

|  | |
| --- | --- |
| If another service was involved **or** you went back to the first or second service again, please answer the questions in **Section D.** | |
|  |  |
| If no-one else was involved, please go to **Section E** on **page 9.** | |
|  | |
|  |  |

| **SECTION D. The third service** | |  | |
| --- | --- | --- | --- |
|  | | | |
| **D1.** What was the **third** service? | | | |
| ***Please tick one*** | | | |
| GP in hours |  | Walk-in Centre |  |
| GP out of hours |  | Minor Injuries Unit |  |
| Accident and Emergency |  | A pharmacist or chemist |  |
| 999 emergency ambulance |  | NHS Direct |  |
| Mental health crisis team |  |  |  |
|  | |  | |
| If the **third** service is not listed, please write it here ________________________________ | | | |
|  | | | |
|  | |  | |

|  |  | | | |
| --- | --- | --- | --- | --- |
| **D.2** When was help sought? | | | | |
| ***Please tick one*** | | | | |
| Monday to Friday between 8.30 am and 6.00 pm | |  |  |  |
| Monday to Friday outside these hours | |  |  | |
| Saturday or Sunday, any time | |  |  | |
|  | |  |  | |

|  | |  | |
| --- | --- | --- | --- |
| **D.3** How was help sought? | |  | |
| ***Please tick one*** | | | |
| By telephone |  |  |  |
| By the Internet |  |  | |
| In person |  |  | |
|  | | | |
| If how you sought help is not listed, please write it here ____________________________ | | | |
|  | | | |

| **D.4** Did contact with this **third** service cost money,  for example on phone calls, petrol, bus fares, parking, taxi fares, etc. | | | | | **YES** | | | **NO** |
| --- | --- | --- | --- | --- | --- | --- | --- | --- |
|  | | | | | | | | |
|  | | | | | | | | |
| If ‘**YES**’, approximately how much? | | | £ : p | | | | | |
|  | | |  | | | | | |
|  | | | | | | | | |
| **D.5** What help did this **third** service give? | | | | | | | | |
| *Please tick* ***all*** *that apply* | | | | | | | | |
|  | | | | | | | | |
| Treatment |  | Physical examination | | | | |  | |
| Tests |  | Advice to go to another service | | | | |  | |
| Prescription |  | Contacted another service for you | | | | |  | |
| Information |  | Asked you to come back again | | | | |  | |
| Reassurance |  | Nothing | | | | |  | |
|  | | | | | | | | |
| If what was given is not listed, please write it here __________________________________ | | | | | | | | |
|  | | | | | | | | |
|  | | | |  | |  | | |

|  | | | | |
| --- | --- | --- | --- | --- |
| **D.6** After this third service did you try to contact another service to seek help or advice about the same problem? | | | **YES**  **NO** |  |
|  | |  | | |
| If ‘**YES**’, why did you take this action? | | | | |
| *Please tick* ***all*** *that apply* | | | | |
| I was told to do so by the third service |  | The health problem got worse | |  |
| I wanted another opinion |  | The health problem changed | |  |
| I could not get access to the third service |  | I was not satisfied with the response from the third service | |  |
| If your reason(s) are not listed, please write them here | | ______________________________________ | | |
|  | |  | | |

| **SECTION E. Looking back** | | | | | | | | |
| --- | --- | --- | --- | --- | --- | --- | --- | --- |
|  | | | | | | | | |
|  | | | | | | | | |
| **E.1** Did you think your case was managed with sufficient urgency? | | | | | | | | |
| ***Please tick one*** | | | | | | | | |
| Definitely not | |  | | | | |  | |
| No, I don’t think so | |  | | | | |  | |
| Yes, I think so | |  | | | | |  | |
| Yes, definitely | |  | | | | |  | |
|  | | | | | | | | |
|  | | | | | | | | |
| **E.2** How long did it take from the time the first service was contacted until the help you wanted was received? | | | | | | | | |
| days ***OR***  hours ***OR*** minutes | | | | | | | | |
| ***Please tick*** | | | | | | | | |
| ***OR*** Did not receive the help required | | | | | | | | |
|  | | | | | | | | |
|  | | | | | | | | |
| **E.3** How do you feel about the number of services contacted? | | | | | | | | |
|  | | | | ***Please tick one*** | | | | |
| Too many services |  | | Too few services | |  | The right number of services | |  |
|  | | | | | | | | |

| **E.4** Here are some statements about **GETTING SERVICES**. Please **tick** one box on each line: | | | | | | | | | |
| --- | --- | --- | --- | --- | --- | --- | --- | --- | --- |
|  | **Strongly agree** | **Agree** | | **Not sure** | | **Disagree** | | **Strongly disagree** | |
|  |  | |  | |  | |  | |  |
| **a.** I did not know which service to go to about this problem |  | |  | |  | |  | |  |
| **b.** I felt that the first service I tried was the right one to help me |  | |  | |  | |  | |  |
| **c.** I felt sometimes I had ended up in the wrong place |  | |  | |  | |  | |  |
| **d.** I found it easy to get the service I wanted |  | |  | |  | |  | |  |
| **e.** Travelling to the services I needed was easy |  | |  | |  | |  | |  |
| **f.** I had to push to get the help I needed |  | |  | |  | |  | |  |

| **E.5** Here are some statements about **THE WAY SERVICES HELPED YOU.** Please **tick** one box on each line: | | | | | | | | | |
| --- | --- | --- | --- | --- | --- | --- | --- | --- | --- |
|  | **Strongly agree** | **Agree** | | **Not sure** | | **Disagree** | | **Strongly disagree** | |
|  |  | |  | |  | |  | |  |
| **a.** I moved through the system smoothly |  | |  | |  | |  | |  |
| **b.** It took too long to get the care needed |  | |  | |  | |  | |  |
| **c.** I felt that no one took responsibility and sorted out my problem |  | |  | |  | |  | |  |
| **d.** I saw the right people |  | |  | |  | |  | |  |
| **e.** I felt I was given the wrong advice |  | |  | |  | |  | |  |
| **f.** My problem was sorted out |  | |  | |  | |  | |  |
| **g.** I got the help I wanted quickly |  | |  | |  | |  | |  |

| **E.6** Here are some statements about **COMMUNICATION.** Please **tick** one box on each line: | | | | | | | | | |
| --- | --- | --- | --- | --- | --- | --- | --- | --- | --- |
|  | **Strongly agree** | **Agree** | | **Not sure** | | **Disagree** | | **Strongly disagree** | |
|  |  | |  | |  | |  | |  |
| **a.** I had to repeat myself too many times |  | |  | |  | |  | |  |
| **b.** Services had the information they needed about me |  | |  | |  | |  | |  |
| **c.** Services did not seem to talk to each other |  | |  | |  | |  | |  |
| **d.** I was told how long I’d have to wait |  | |  | |  | |  | |  |
| **e.** My concerns were taken seriously by everyone |  | |  | |  | |  | |  |

| **E.7** Here are some statements about things **IN GENERAL.** Please **tick** one box on each line: | | | | | | | | | |
| --- | --- | --- | --- | --- | --- | --- | --- | --- | --- |
|  | **Strongly agree** | **Agree** | | **Not sure** | | **Disagree** | | **Strongly disagree** | |
|  |  | |  | |  | |  | |  |
| **a.** Services understood that I had responsibilities, like my need to look after my family |  | |  | |  | |  | |  |
| **b.** I was made to feel like I was wasting everyone’s time |  | |  | |  | |  | |  |
| **c.** The system did not work well this time |  | |  | |  | |  | |  |
| **d.** At each stage, I was confident in the advice services gave me |  | |  | |  | |  | |  |

|  | | | |  | |
| --- | --- | --- | --- | --- | --- |
| **E.8** Overall, how would you rate the care you received? | | | |  | |
| ***Please tick one*** | | | | | |
| Excellent |  |  | | |  |
| Very good |  |  | | |  |
| Good |  |  | | |  |
| Fair |  |  | | |  |
| Poor |  |  | | | |
| Very poor |  |  | | | |
|  | | |  | | |

| **E.9 In the last 3 months, about how many times has help been sought for an urgent health problem?**  times  If ‘**MORE THAN ONCE**’, was the most recent time……  ***Please tick one***  Much the same as other times  Better  Worse |
| --- |

| **SECTION F. How can local services be improved when help or advice is needed for an urgent health problem?** |
| --- |
|  |
|  |
|  |
|  |
|  |
|  |
|  |
|  |
|  |
|  |
|  |
| **Any other comments?** |
|  |
|  |
|  |
|  |
|  |
|  |
| **THANKS FOR YOUR HELP** |
| **Please use the FREEPOST envelope provided to send the questionnaire back** |
|  |
| **If you would like to talk to a member of the research team**  **about this questionnaire, please ring**  **0114 2220850** |
|  |
|  |

Medical Care Research Unit, University of Sheffield, Regent Court, Regent Street

Sheffield S1 4DA
